# Supplementary material for: Flexible power generators by Ag2Se thin films with record-high thermoelectric performance
Source: Nat Commun. 2024 Jan 31;15:923. doi: 10.1038/s41467-024-45092-7 (PMC10830499; doi:10.1038/s41467-024-45092-7)
Supplement: Supplementary file 1 — Supplementary Information [file 41467_2024_45092_MOESM1_ESM.pdf]

Supplementary information

Flexible power generators by Ag_2Se thin films with record-high thermoelectric performance

Dong Yang,[1,2,a] Xiao-Lei Shi,[3,a] Meng Li,[3] Mohammad Nisar,[1] Adil Mansoor,[1] Shuo Chen,[1] Yuexing Chen,[1] Li Fu,[1] Hongli Ma,[2] Guang Xing Liang,[1] Xianghua Zhang,[2] Weidi Liu,[3,4] Ping Fan,[1] Zhuanghao Zheng,[1,] and Zhi-Gang Chen[3,*]*

[1] Shenzhen Key Laboratory of Advanced Thin Films and Applications, Key Laboratory of Optoelectronic Devices and Systems of Ministry of Education and Guangdong Province, College of Physics and Optoelectronic Engineering, Shenzhen University, Shenzhen, Guangdong 518060, China;

[2] Univ Rennes, CNRS, ISCR (Istitut des Sciences Chimiques de Rennes) UMR 6226, Rennes, F-35000, France;

[3] School of Chemistry and Physics, ARC Research Hub in Zero-emission Power Generation for Carbon Neutrality, and Centre for Materials Science, Queensland University of Technology, Brisbane, Queensland 4001, Australia;

[4] Australian Institute for Bioengineering and Nanotechnology, The University of Queensland, Brisbane, QLD 4072, Australia;

[a] These authors contribute equally to this work.

Corresponding Author

* Zhuanghao Zheng: zhengzh@szu.edu.cn;

* Zhi-Gang Chen: zhigang.chen@qut.edu.au.

1. Supplementary Experimental details

Thin film and device fabrication. Flexible Ag_2Se thin films were deposited using the vacuum thermal co-evaporation method at various temperatures. High-purity Ag powders (99.99% purity, Aladdin) and Se-Te mixed powders (99.99% purity, Aladdin) were loaded into a vacuum deposition chamber using tantalum evaporator boats. A polyimide (PI) substrate measuring 20 mm × 20 mm × 2 mm was cleaned ultrasonically for 15 minutes consecutively in acetone, ethanol, and deionized water. The background pressure in the vacuum deposition chamber was maintained at 5.4×10^{-4} Pa. The silver source had an evaporating current of 130 A, while the selenium source stabilized at 40 A, with a concurrent deposition time of 15 minutes. The chemical composition of the samples was controlled by adjusting the mass ratio. All Ag and Se powders were evaporated after the preparation process. To address the issue of insufficient atomic energy during room temperature deposition, the substrate was heated to 353 K during deposition. The average film thickness was 350 nm. A single Ag_2Se-Sb_2Te_3 thermoelectric device was fabricated with a Cu electrode on a PI substrate. The legs had fixed dimensions of 20 mm × 4 mm. An organic coating material, polyvinyl laurate, was employed to enhance the flexibility of the as-fabricated Ag_2Se thin films. First, the Ag_2Se thin films were securely laid flat on glass. Second, an appropriate amount of polyvinyl-laurate-based polymers composed of 90% poly(vinyl laurate) and 10% N-methylpyrrolidone was applied to the thin films under vacuum conditions. The thin films were then placed in a centrifuge for 10 seconds with a rotation speed of 2000 r/s to ensure uniform coverage of the organic layer. Subsequently, the samples were heated to 95 °C for 2 minutes and exposed to ultraviolet light for 15 seconds.

Characterizations and thermoelectric performance evaluation. X-ray diffraction (XRD) analysis was performed using a Rigaku Corporation D/max 2500 instrument with CuKα radiation over an angular

range of 20° to 60° in 0.02° increments to determine the crystal orientation of the Ag_2Se samples. The preferred orientation information of the Ag_2Se thin films was obtained through pole figure scans using a four-circle X-ray diffractometer (X'PERT, Philips). Morphological analysis, mapping, and composition assessment of the samples were conducted using a Zeiss Supra55 scanning electron microscope (SEM). An energy dispersive X-ray (EDS) detector from Bruker, known as the EDS QUANTAX, was used for EDS analysis. The electrical conductivity σ and Seebeck coefficient S were measured using a Seebeck coefficient and electrical conductivity apparatus (SBA458, Netzsch). The in-plane thermal conductivity κ of the thin film in this work, deposited on a commercial flat chip, was determined using the 3ω method through a thin film comprehensive physical property analyzer (TFA-LINSES). Carrier concentration n and mobility μ were investigated using a Van der Pauw Hall measuring instrument (HL5500 PC, Nanometrics). Contact resistivity ρ_c was measured using a 2400 series digital source meter.

Preferred orientation factor calculation: The orientation factor F was calculated by the equations $F = \frac{P-P_0}{1-P_0}$, $P = \frac{I(00l)}{\Sigma I(hkl)}$, and $P_0 = \frac{I_0(00l)}{\Sigma I_0(hkl)}$, where $I(00l)$ is the sum of the diffraction intensity of $(00l)$ planes, $\Sigma I(hkl)$ is the total intensity of all (hkl) diffraction peaks and P is the ratio of the intensity of $(00l)$ plane in the measured data. The $I_0(00l)$, $\Sigma I_0(hkl)$, and P_0 represent the corresponding ones from the standard pattern.

Single parabolic (SPB) model calculation: We employed an SPB model to perform a simulation to understand our measured properties by using the following equations [1-4]:

$$S(\eta) = \frac{k_B}{e}.\left[\frac{\left(r+\frac{5}{2}\right).F_{r+\frac{3}{2}}(\eta)}{\left(r+\frac{3}{2}\right).F_{r+\frac{1}{2}}(\eta)} - \eta\right] \tag{1}$$

$$n_H = \frac{1}{e.R_H} = \frac{(2m^*.k_B T)^{\frac{3}{2}}}{3\pi^2\hbar^3}.\frac{\left(r+\frac{3}{2}\right)^2.F_{r+\frac{1}{2}}{}^2(\eta)}{\left(2r+\frac{3}{2}\right).F_{2r+\frac{1}{2}}(\eta)} \tag{2}$$

$$\mu_H = \left[\frac{e\pi\hbar^4}{\sqrt{2}(k_BT)^{\frac{3}{2}}}\frac{C_1}{E_{def}^2(m^*)^{\frac{5}{2}}}\right]\frac{\left(2r+\frac{3}{2}\right).F_{2r+\frac{1}{2}}(\eta)}{\left(r+\frac{3}{2}\right)^2.F_{r+\frac{1}{2}}(\eta)} \quad (3)$$

$$F_j(\eta_F) = \int_0^\infty \frac{x^j}{1+e^{x-\eta_F}}dx \quad (4)$$

where j is the Fermi integral of order, η_F and $\hbar$ are reduced Fermi level (E_f/k_BT) and Plank constant. By using the experimentally measured S and n in the above formula and assuming acoustic phonon scattering is prominent (*i.e., $r = -1/2$*), the theoretical transport properties can be calculated.

Computational calculation: First-principles calculations were performed based on density-functional theory (DFT) with all electron projected augmented wave (PAW) method, as implemented in the Vienna Ab initio Simulation Package (VASP) [5-10]. Semi-local generalized gradient approximation (GGA) with the fully relativistic Perdew-Burke-Ernzerhof (PBE) exchange correlation functional was employed [11]. The valence wave functions were expanded in a plan-wave basis with a cut-off energy of 400 eV. To simulate doping of Te, a 1×1×3 supercell containing 36 atoms was created, where one of the 12 Se atoms was substituted by one Te atom. All atoms were allowed to relax in their geometric optimizations, with the Brillouin zone sampled by a Monkhorst-Pack **k**-mesh of 2×5×4 (4×7×6 for self-consistent calculations). The convergence criterion was set to be 1×10^{-7} eV per electron and 1×10^{-3} eV·Å^{-1} per unit cell. The Hubbard U model was included in non-self-consistent calculations, with the on-site coulombic (U) and the exchange (J) terms combined in a single effective U parameter of 5.8 eV for Ag_4d orbitals, determined based on linear response theory [12]. Because Te is a heavy element, the spin-orbital coupling (SOC) effect was considered for the calculation of band structures, which is projected on the **k**-path indicated by the AFLOW framework [13,14].

For the calculations of (001) surface energy variation before and after Te-doping, the spin-polarized density functional theories (DFT) were performed by using the Vienna Ab initio Simulation Package (VASP) [7]. The Perdew-Burke-Ernzerhof generalized-gradient approximation functional was

used to describe the interaction between electrons [11]. The energy cutoff was set to 400 eV. The Monkhorst-Pack k-points were set to be $6 \times 4 \times 1$ during the calculations. The vacuum region was set to be 15 Å in the z-direction to prevent the interaction between two adjacent surfaces. The energy convergence and atomic forces were set to 10^{-5} eV and 0.02 eV/Å. The surface energy γ_s, is the energy required to cleave a surface from the bulk phase, which describes the stability of the surface. The γ_s is calculated by the below equation [15]:

$$\gamma_s = \frac{1}{2A}(E_{slab} - NE_{atom}) \quad (5)$$

Here A is the area of the surface. E_{slab} is the energy of the slab. N is the number of metal atoms. E_{atom} is the energy for one atom (Ag, Se, and Te) referred to the corresponding bulk structures.

For the calculations of (002) surface energy variation before and after Te-doping, the commercial software package Material Studio, including the Cambridge Serial Total Energy Package (CASTEP) module was used to conduct this geometric optimization to determine the initial lattice energy. In the process, the generalized-gradient approximation (GGA-PBE) along with OTFG ultrasoft pseudo potentials was used to perform the geometric optimization. The (002) surface was created in Material Studio software, the cleave rule is default and the vacuum thickness is 15Å.

Debye Callaway model calculations: Debye Callaway model is investigated to clarify the source of reduction in κ_l [16]:

$$\kappa_l = \frac{4\pi {k_B}^4 T^3}{vh^3} \int_0^{\frac{\theta_D}{T}} \tau_T \frac{\chi^4 \exp(\chi)}{[\exp(\chi)-1]^2} d\chi \quad (6)$$

here v, θ_D, γ, and τ_T stand for sound velocity, Debye temperature, reduced phonon frequency ($\chi = \hbar\omega/k_B T$), and total phonon relaxation time.

The phonon-scattering phenomenon involves different scattering progressions from electron-phonon interaction (τ_{EP}^{-1}), dislocations (τ_{DS}^{-1}), impurity/point defects (τ_{PD}^{-1}), grains (τ_{GB}^{-1}), and Umklapp-

scattering (τ_U^{-1}), in the doped system. Hence, τ_T for doped Ag_2Se is demonstrated as [17]:

$$\frac{1}{\tau_T} = \frac{1}{\tau_U} + \frac{1}{\tau_N} + \frac{1}{\tau_{PD}} + \frac{1}{\tau_{DS}} + \frac{1}{\tau_{GB}} \tag{7}$$

where τ_U, τ_N, τ_{PD}, τ_{IF}, τ_{NP}, τ_{DS}, and τ_{SF} are relaxation time corresponding to the scattering from phonon-phonon U- and N-process (U+N), vacancies/alloy elements (point defects, PD), grain boundaries (GB), and dislocations (DS). As a result, τ_T (the total phonon relaxation time) for the doped system shows a significant reduction as compared to τ_T for the undoped Ag_2Se system due to the existence of intense scattering effects under alloying or doping.

Further, Debye-Callaway model-based spectral lattice thermal conductivity κ_s was derived for doped samples to reveal the influence of all possible phonon scattering mechanisms, as follows [18]:

$$\kappa_S = \frac{4\pi\, {k_B}^4\, T^3}{V_s h^3}\, \tau_T \frac{z^4 \exp(z)}{[\exp(z)-1]^2} \tag{8}$$

In the present doped system, the main τ_T reduction is related to the phonon-scattering phenomenon from scattering due to impurity/point defects (τ_{PD}^{-1}), and grain boundaries (τ_{GB}^{-1}),

Hereafter, the phonon scattering from prominent scattering centers can be simplified as [19]:

$$\tau_{PD}^{-1} = \frac{V\,\Gamma}{4\pi v^3}\omega^4 \propto A\omega^4 \tag{9}$$

$$\tau_U^{-1} = \frac{\hbar\gamma^2\omega^2 T}{M v^2 \theta_D} exp\left(-\frac{\theta_D}{3T}\right) \propto B\omega^2 \tag{10}$$

$$\tau_{EP}^{-1} = \beta\tau_U^{-1} = \beta\frac{\hbar\gamma^2\omega^2 T}{M v^2 \theta_D} exp\left(-\frac{\theta_D}{3T}\right) \propto C\omega^2 \tag{11}$$

$$\tau_{DS}^{-1} = 0.6 B_D^2 N_D \omega\gamma^2 \left\{\frac{1}{2} + \frac{1}{24}\left(\frac{1-2r}{1-r}\right)^2 \left[1 + \sqrt{2}\left(\frac{v_L}{v_T}\right)\right]\right\} + N_D\left(\frac{v^{-4/3}}{v_S^2}\right)\omega^3 \tag{12}$$

The simplified presentation of scattering phenomena via introducing pre-factors is described as follows:

$$\tau_U^{-1} + \tau_N^{-1} = Z_1\omega^2 T \exp\left(-\frac{\theta_D}{3T}\right) \tag{13}$$

$$\tau_{PD}^{-1} = Z_2\omega^4 \tag{14}$$

$$\tau_{\mathrm{DS}}^{-1} = Z_3\omega^1 + Z_4\omega^3 \tag{15}$$

The proposed pre-factors Z_1, Z_2, Z_3, and Z_4 correspond to the scattering from phonon-phonon U- and N-process, vacancies/anti-sites (point defects), and dislocation defects, respectively. The values of the average v is 1462 ms^{-1}, and the θ_D is 63.72 K, both taken from the literature [16].

2. Supplementary Figures

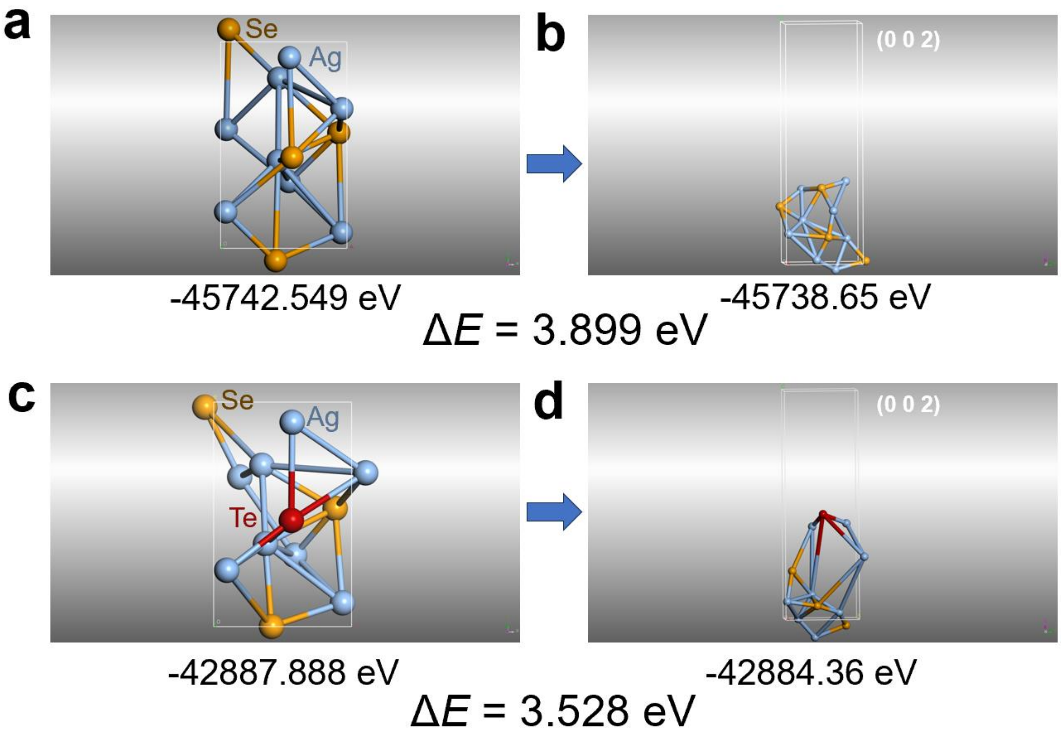

Supplementary Fig. 1. (**a**) Unit cell of pristine Ag_2Se. (**b**) Unit cell of pristine Ag_2Se cut along the (002) surface. The vacuum layer thickness is 15 Å. (**c**) Unit cell of Te-doped Ag_2Se. (**d**) Unit cell of Te-doped Ag_2Se cut along the (002) surface. The vacuum layer thickness is 15 Å. Here, the undoped Ag_2Se unit cell energy is -45742.549 eV, and the undoped Ag_2Se unit cell energy after cutting along the (002) crystal plane is -45738.65 eV, therefore the energy difference ΔE = 3.899 eV. Oppositely, the Te-doped Ag_2Se unit cell energy is -42887.888 eV, while the Te-doped Ag_2Se unit cell energy after cutting along the (002) crystal plane is -42884.36 eV, therefore the ΔE = 3.528 eV, which is smaller than 3.899 eV, indicating that the introduction of Te on Se sites is beneficial for the formation of the (002) surface of Ag_2Se.

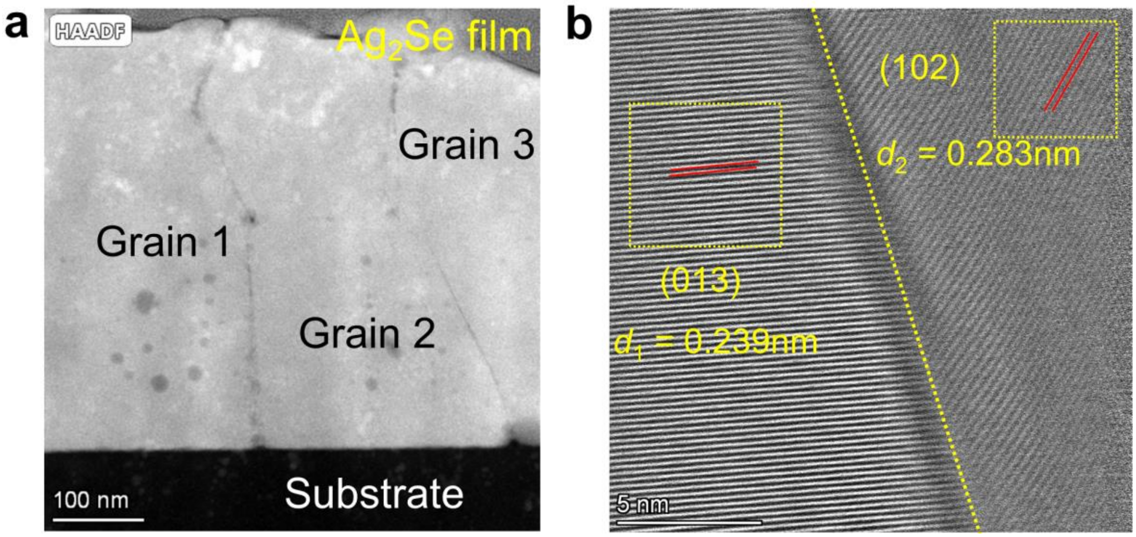

Supplementary Fig. 2. (**a**) Low-magnification transmission electron microscopy (TEM) high-angle annular dark field (HAADF) image of pristine Ag_2Se thin film with anisotropic grains. (**b**) High-resolution TEM (HRTEM) image to show a grain boundary between two grains.

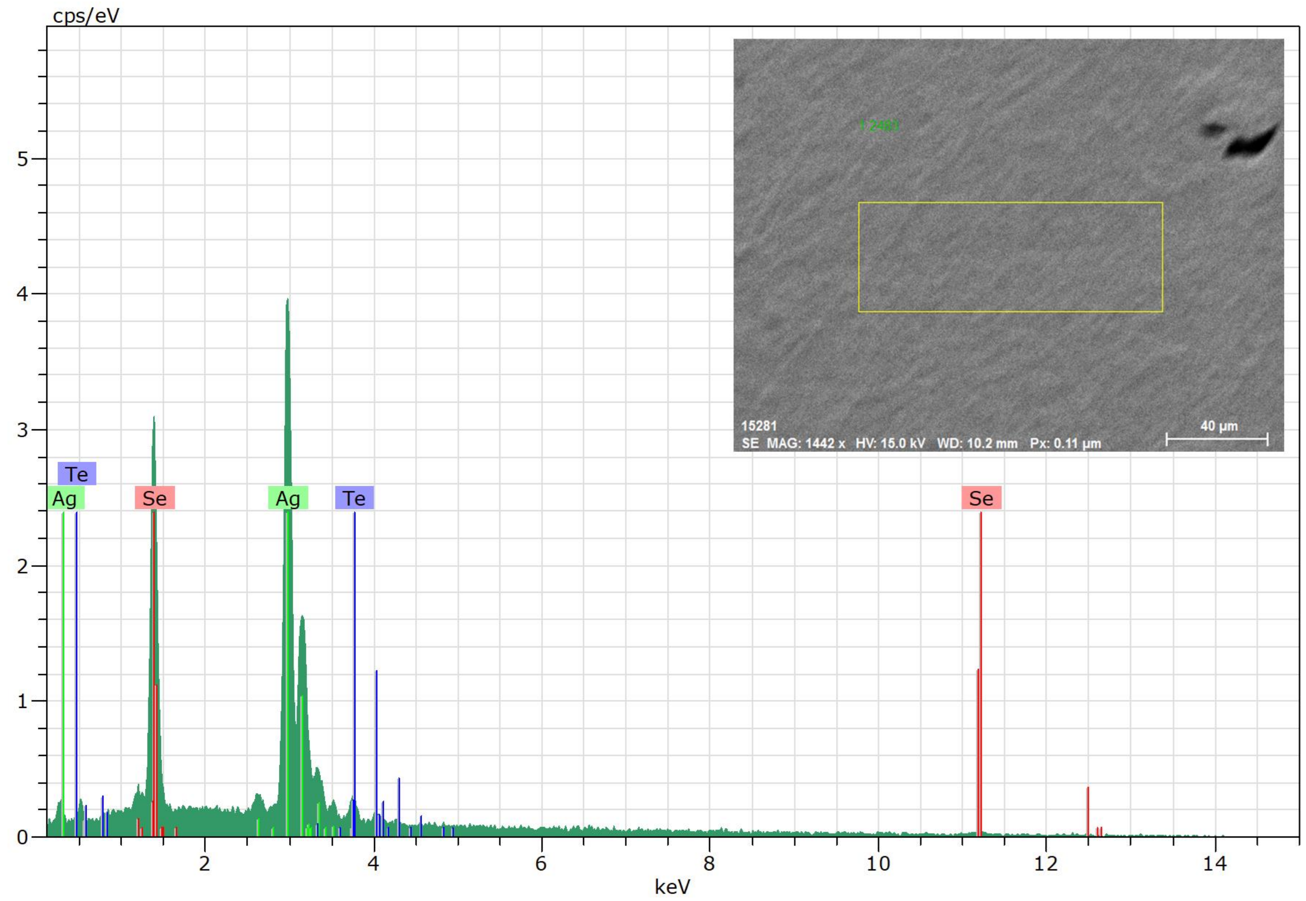

Supplementary Fig. 3. Energy dispersive spectroscopy (EDS) results with inset of scanning electron microscopy (SEM) image for Ag_2Se thin films with 0.7 at.% Te.

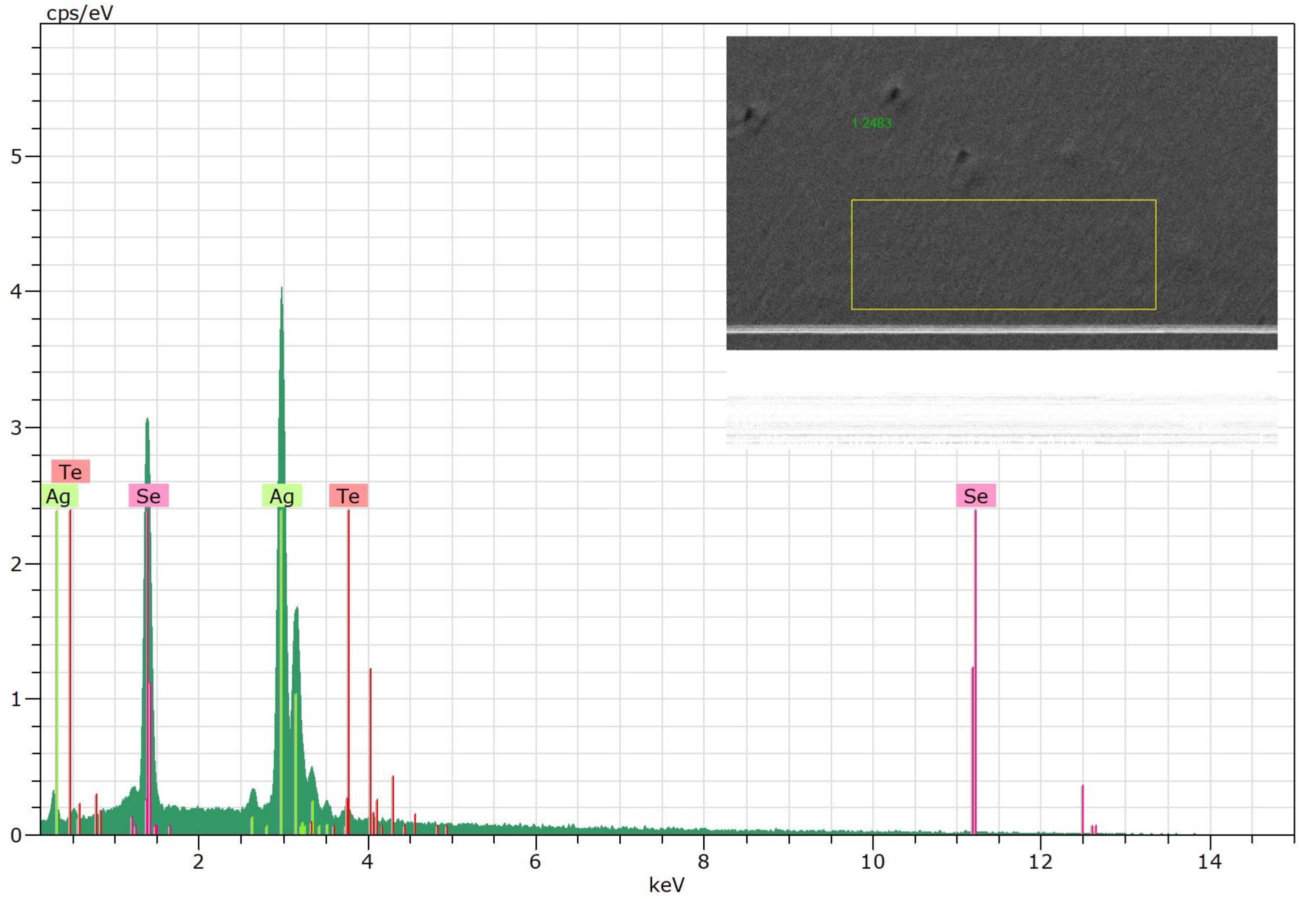

Supplementary Fig.4. EDS results with inset of SEM image for Ag_2Se thin films with 1.3 at.% Te.

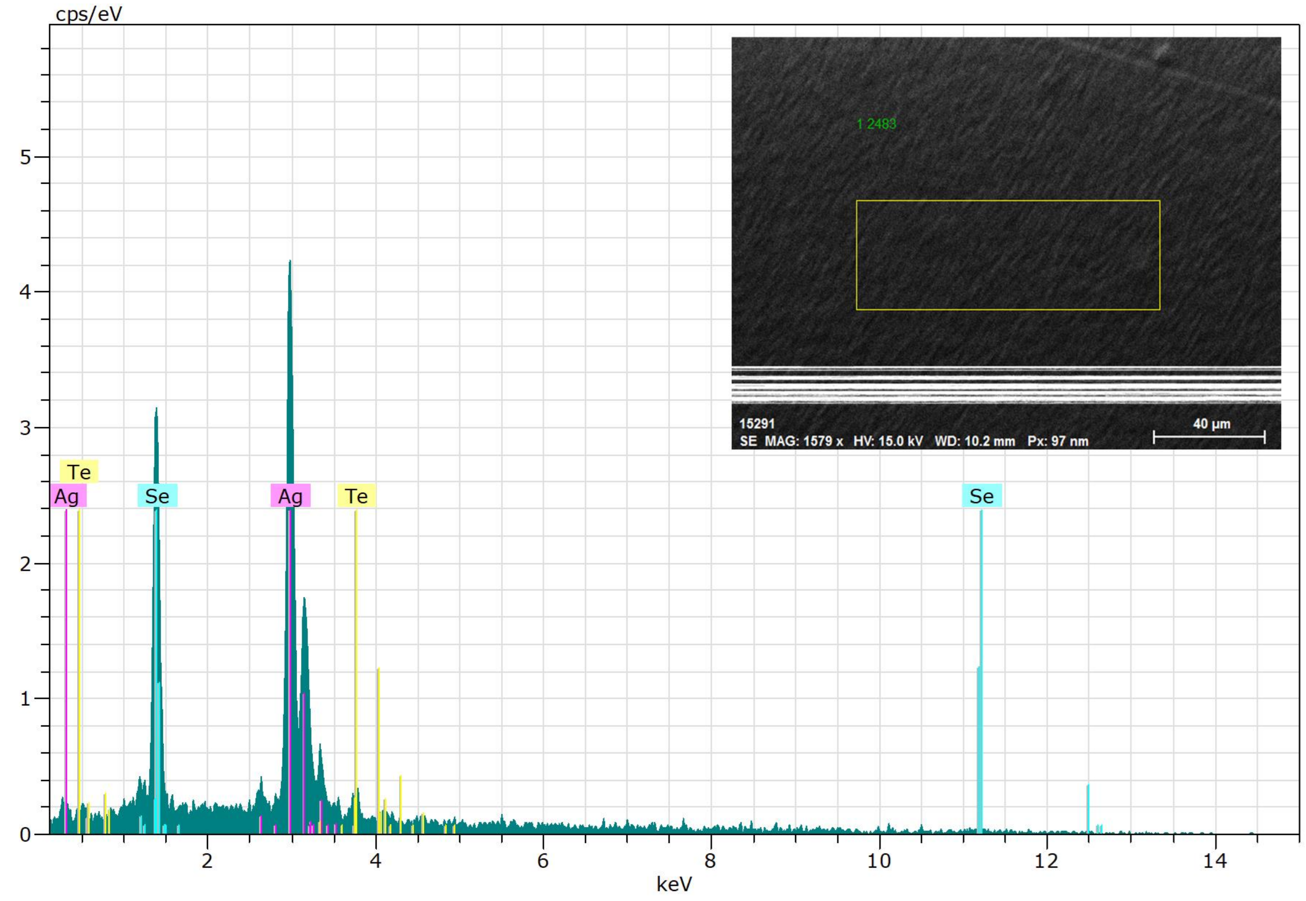

Supplementary Fig. 5. EDS results with inset of SEM image for Ag_2Se thin films with 1.9 at.% Te.

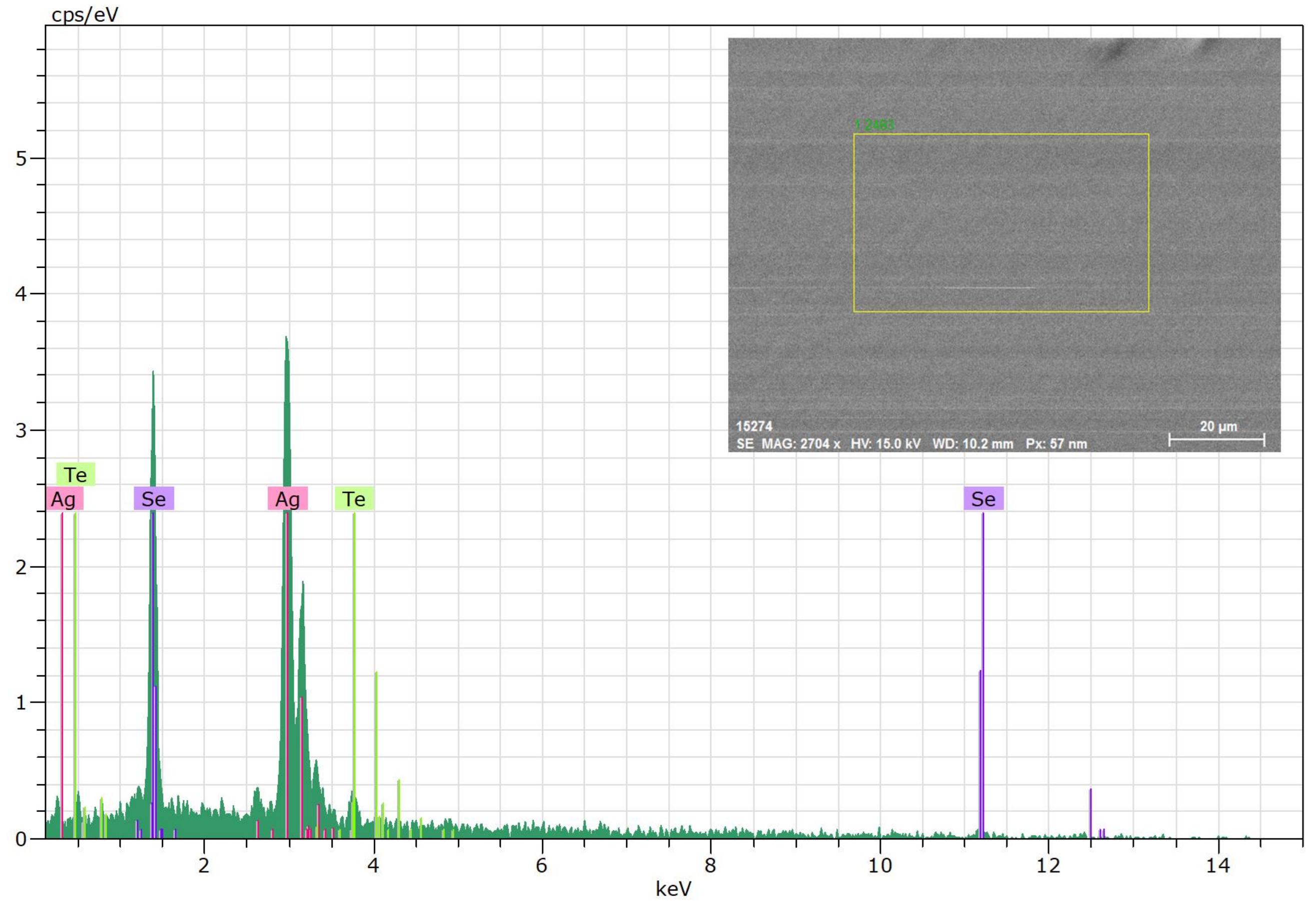

Supplementary Fig. 6. EDS results with inset of SEM image for Ag_2Se thin films with 2.6 at.% Te.

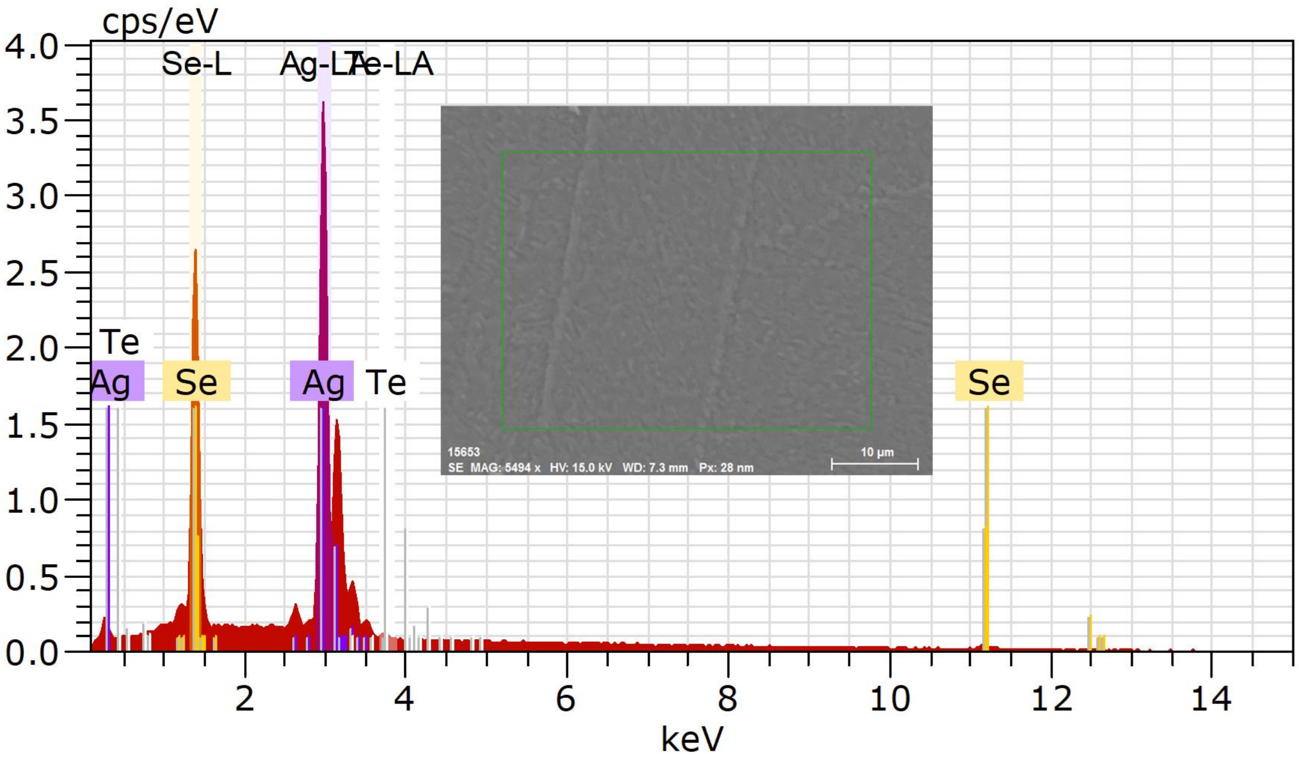

Supplementary Fig. 7. EDS results with inset of SEM image for Ag_2Se thin films with 3.8 at.% Te.

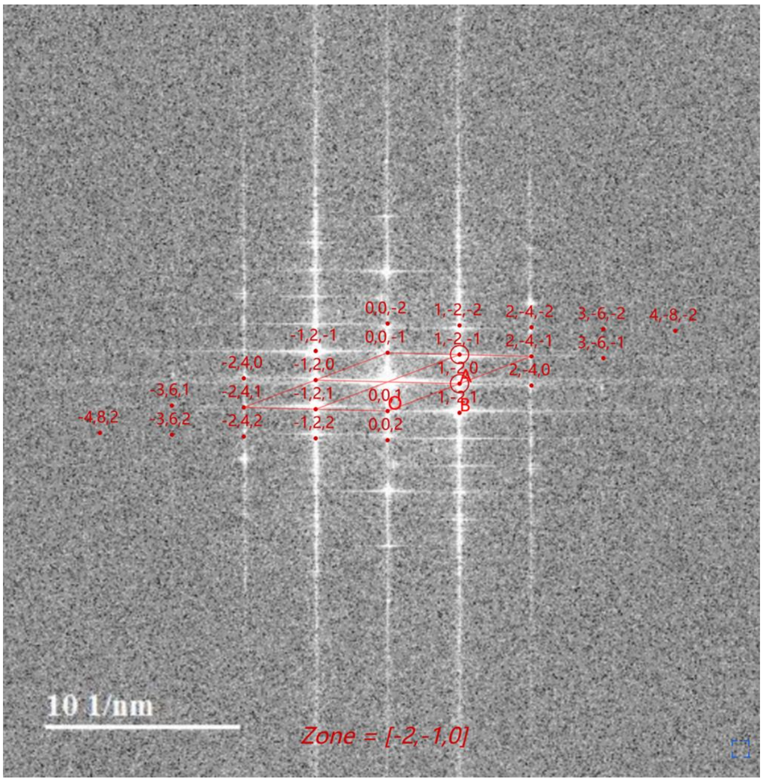

Supplementary Fig. 8. Magnified indexed fast Fourier transform (FFT) pattern taken from **Figure 3e** in the manuscript.

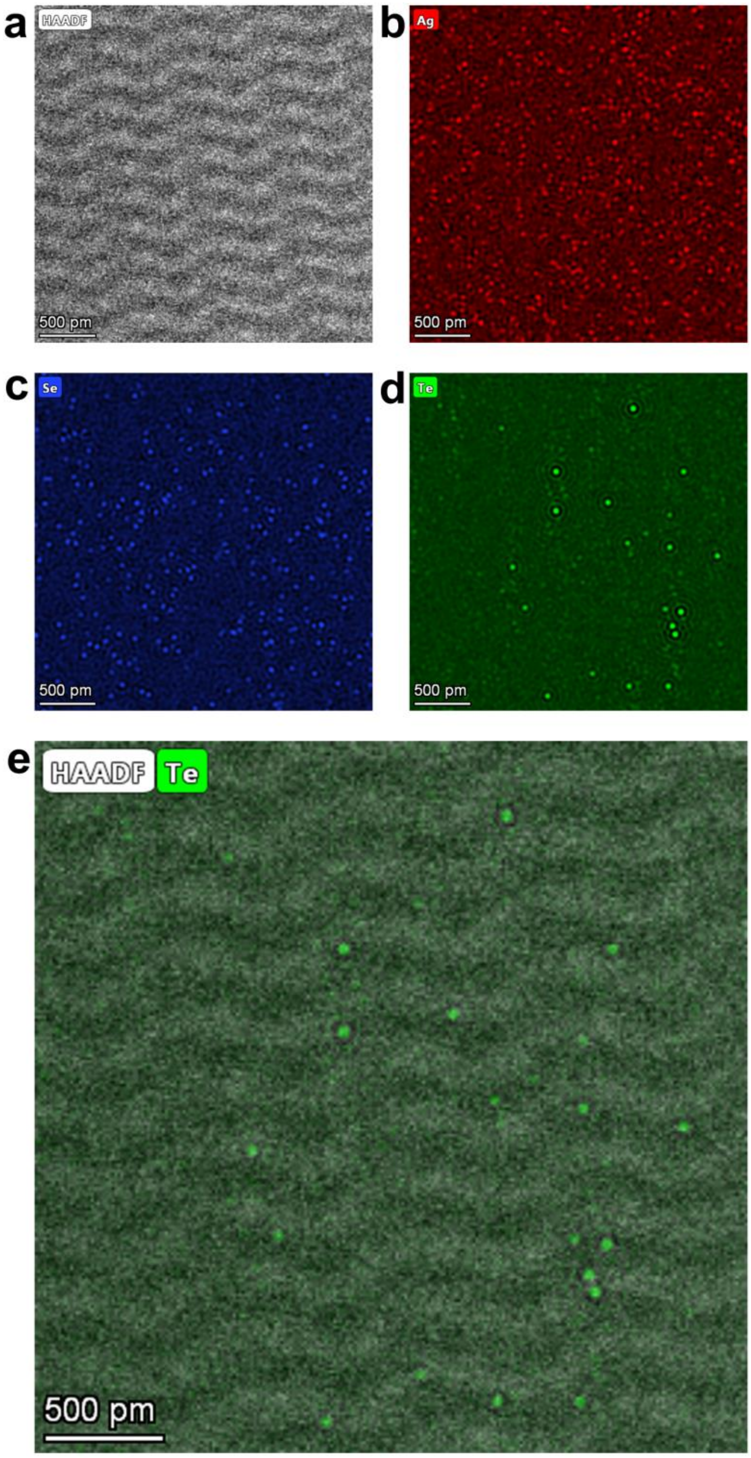

Supplementary Fig. 9. (**a**) Spherical aberration-corrected scanning TEM (Cs-STEM) HAADF image of Ag_2Se thin film with 3.2 at.% Te, and corresponding EDS maps of (**b**) Ag, (**c**) Se, and (**d**) Te. (**e**) Overlap of the HAADF image and the EDS map of Te.

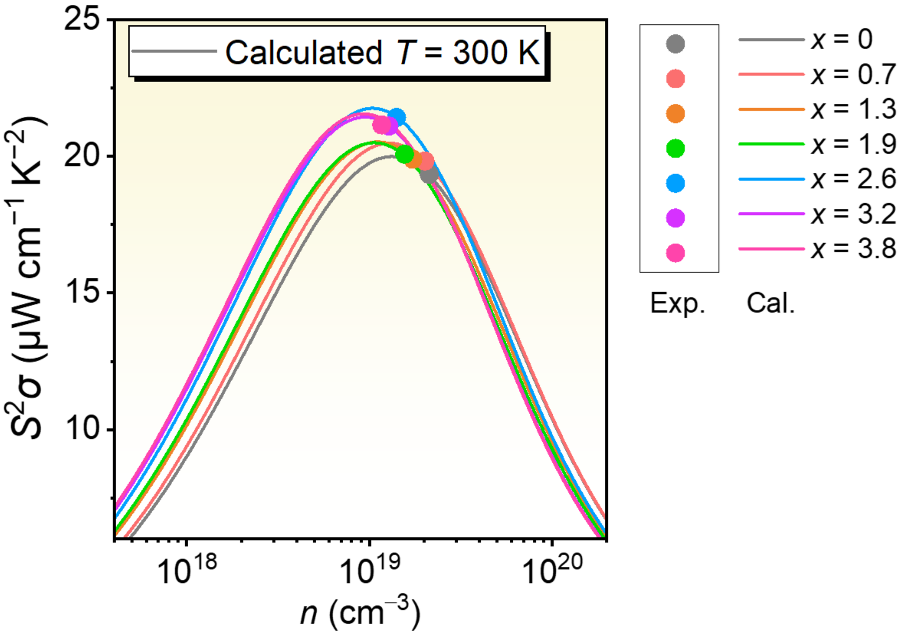

Supplementary Fig. 10. Comparison of calculated and experimental power factor $S^2\sigma$ as a function of carrier concentration n. The calculated data are from the single parabolic band (SPB) model.

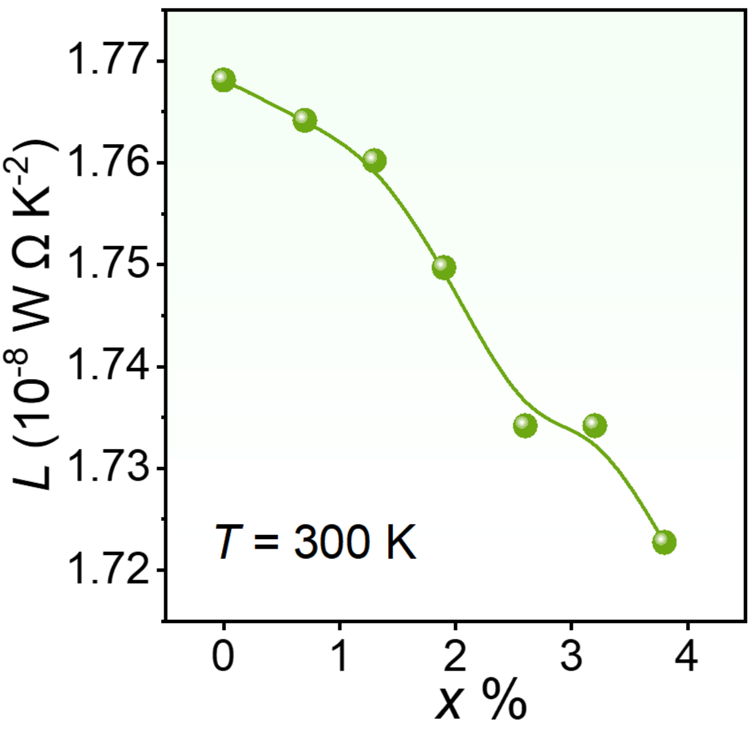

Supplementary Fig. 11. Te concentration *x*-dependent Lorenz number *L* at room temperature.

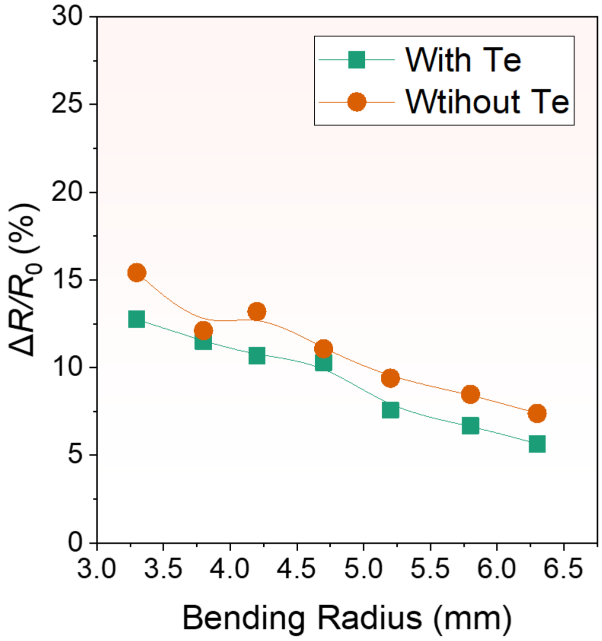

Supplementary Fig. 12. Measured increased normalized resistance $\Delta R/R_0$ of the 3.2 at.% Te-doped and undoped Ag_2Se thin films at different bending radii.

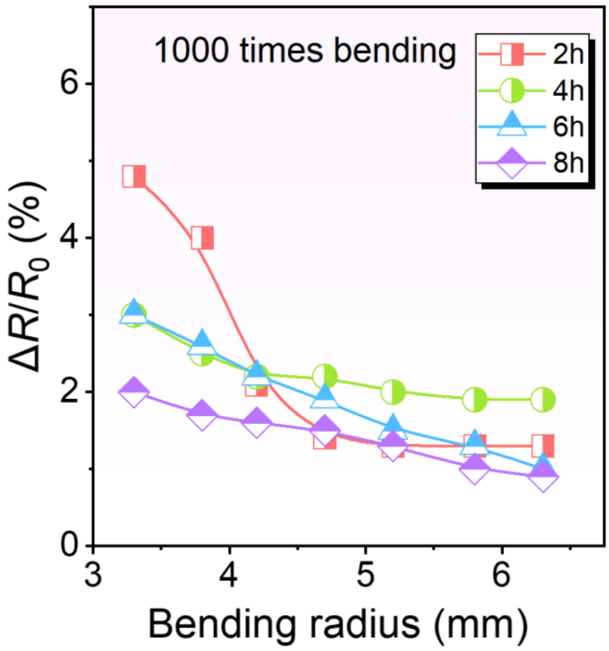

Supplementary Fig. 13. Measured increased normalized resistance $\Delta R/R_0$ of the coated Ag_2Se thin films with 3.2 at.% Te at different bending radii and heating at 95 °C by different periods for 1000 times bending.

Measurement method: Transmission line measurement (TLM)

Measurement tool: 2400 Series Digital Source-meter

Electrode: 3 mm x 1.5 mm

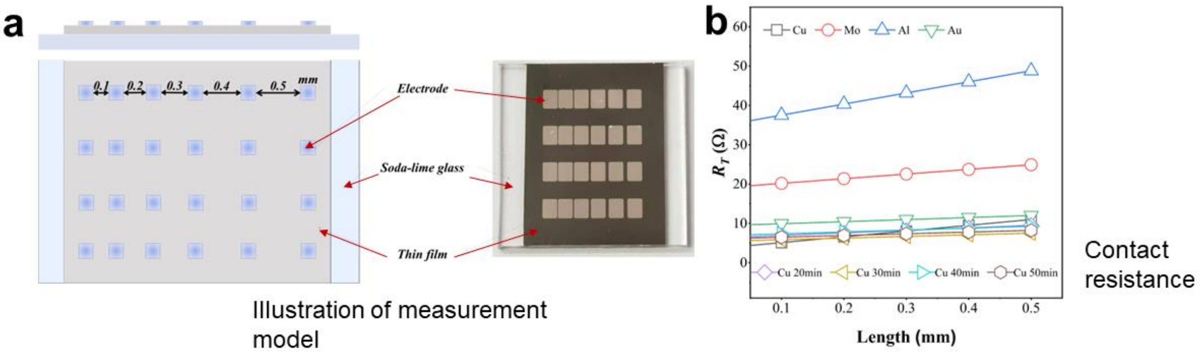

Supplementary Fig. 14. (**a**) Illustration of measurement model for evaluating the contact resistance. Reproduced with permission [20]. Copyright 2022, Elsevier. (**b**) As-measured contact resistance as a function of length for different electrode materials.

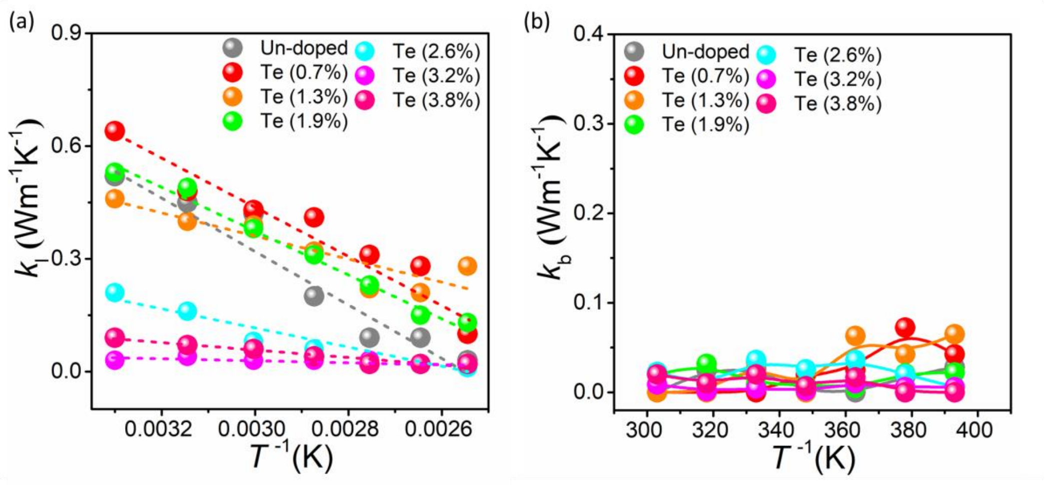

Supplementary Fig. 15. (a) The dotted line is linearly fitting to the lattice thermal conductivity (κ_l) at low temperature. (b) Bipolar thermal conductivity (κ_b) for Ag_2Se thin films with different Te concentrations (x = 0, 0.7, 1.3, 1.9, 2.6, 3.2, and 3.8 at.%)

3. Supplementary Table

Supplementary Table 1. A summary of the thermoelectric performance of Ag_2Se-based films. Here, poly(3,4-ethylenedioxythiophene) is abbreviated as PEDOT; polyvinylidene fluoride is abbreviated as PVDF; polyvinylpyrrolidone is abbreviated as PVP; single wall carbon nanotubes is abbreviated as SWCNTs; polypyrrole is abbreviated as PPy; polypropylene is abbreviated as PI; polyethylene naphthalate two formic acid glycol ester is abbreviated as PEN; and polyethylene terephthalate is abbreviated as PET.

**Materials**	**Substrate**	***ZT***	***T* (K)**	***S* (μV K^{-1})**	***σ* (S cm^{-1})**	**$S^2\sigma$ (μW cm^{-1} K^{-2})**	***κ* (W m^{-1} K^{-1})**	***n* (cm^{-3})**	***μ* (cm^2 V^{-1} s^{-1})**	**Year**	**Ref.**
Ag_2Se thin film with 3.2 at.% Te	PI	**1.27**	363	–132	1425	24.8	0.71	1.28×10^{19}	444	2023	This work
Ag_2Se	PI	~1.2	300	–135	~1440	~25.9	~0.66	6.5×10^{18}	1250	2022	[21]
Ag_2Se+PVP	Nylon	1.1	300	–144	925	19.1	/	7×10^{18}	1100	2020	[22]
Ag_2Se	PI	1.1	300	–161.7	840	22.05	0.61	7.3×10^{18}	721.3	2022	[23]
Printed-Ag_2Se	PEN	1.03	300	–185	460	17	0.5	/	/	2020	[24]
Ag_2Se	Scaffold	~1	300	–183	472	15.8	~0.47	/	/	2020	[25]
Ag_2Se+Se+PPy	Nylon	0.94	300	–144	1064	22.4	/	9.5×10^{18}	762	2021	[26]
Ag_2Se+bacteria cellulose	/	0.7	400	–167	230	6.24	0.36	/	/	2022	[27]
Ag_2Se+nylon	Substrate-free	0.68	300	–137.	958.9	18.25	/	~1.2×10^{19}	~480	2022	[28]

				9							
Ag_2Se	Nylon	0.6	300	–141	497	9.87	/	3.8×10^{18}	850	2019	29
$Ag_{2.02}Se$	PI	~0.6	348	~–110	~1785	21.6	~1.2	/	/	2022	30
Printed Ag-Se film	Glass	0.55	300	–220	~108	~5	/	/	/	2020	31
Ag_2Se	Substrate-free	0.5	300	–120	1060	~15.3	~0.89	4.79×10^{18}	1390	2023	32
Ag_2Se	Glass	/	376	–216	974	~46.6	/	1×10^{19}	634	2017	33
$Ag_{2.2}Se$	PI	/	300	–107	3010	~34.2	~0.56	2.5×10^{19}	750	2021	34
$Ag_{2.3}Se$	Paper	/	303	–122	~1660	~24.5	/	2×10^{19}	~415	2020	35
Ag_2Se+Ag+CuAgSe	Nylon	/	300	–45	1050	22.3	/	2.7×10^{20}	250	2019	36
Ag_2Se	PI	/	320	–142	1091	21.9	/	/	/	2023	37
Cu-doped Ag_2Se	PI	/	300	–125	~1350	20.8	/	8.6×10^{18}	~900	2022	38
S-doped Ag_2Se	PET	/	300	–150	~900	20.58	/	$\sim1\times10^{19}$	~570	2023	39
Ag_2Se	Nylon	/	300	–150	~908	20.43	/	5.82×10^{18}	1277	2022	40
Ag_2Se +SWCNTs	Nylon	/	300	–108.1	~1657	~19.4	/	1.31×10^{19}	/	2023	41
$Ag_{1.8}Se$	PI	/	380	–138	1000	19	/	7.5×10^{18}	950	2021	42
Ag_2Se	Nylon	/	300	–143	~920	~18.8	/	7.9×10^{18}	1024	2020	16
Ag_2Se+Ag	Nylon	/	300	–67.5	3958	~18.6	/	1.6×10^{20}	~83	2021	17
Ag_2Se	Glass	/	300	–120	~1200	17.3	/	1×10^{19}	714	2016	18

Ag_2Se+CuAgSe + PEDOT	Nylon	/	300	–121	~1100	16	~0.46	7.4×10^{18}	940	2020	[19]
In-situ $Ag_{2.09}Se$	/	/	423	–110	~1300	15.3	/	1.5×10^{19}	550	2022	[43]
Ag_2Se+Ag+PEDOT	Nylon	/	300	–49.5	~5957	14.4	/	4.4×10^{19}	78	2021	[44]
Ag_2Se+SWCNTs	Nylon	/	397	–118	~880	~12.4	/	7.8×10^{18}	~700	2021	[45]
$Ag_{1.98}Ga_{0.02}Se$	Nylon	/	300	–115	~880	~11.6	/	/	/	2021	[46]
Ag_2Se	Glass	/	300	–123	~730	11	/	4.8×10^{19}	94	2021	[47]
S-doped Ag_2Se	Nylon	/	300	–106	849	~9.5	/	6×10^{18}	~900	2022	[48]
Ag_2Se+PEDOT	/	/	300	–91	520	~4.3	/	/	/	2022	[49]
Ag_2Se+PVDF	Glass	/	300	–92	~370	~2.8	/	/	/	2020	[50]

Supplementary Table 2. Debye-Callaway model-based pre-factors for all Te-doped Ag_2Se samples.

Parameters	Samples						
x	0	0.1	0.2	0.3	0.4	0.5	0.5
A_1 (10^{-41} s^3)	0.005	0.61	0.56	0.77	18.32	41.17	55.51
A_2 (10^{-18} s/K)	4.71	0.21	0.29	0.47	17.60	50.14	44.50
A_3 (10^{-15} s)	6.44	6.44	6.44	6.44	6.44	6.44	6.44
A_4 (10^{-25} s^2)	0.0	0.002	0.002	0.004	0.021	0.029	0.032
d (nm)	----	300	270	220	180	160	178

Supplementary Table 3. A summary and comparison of the power densities of reported thermoelectric flexible devices.

Device materials	Output power density (mW cm^{-2})
Cu_3Se_1 single leg [51]	~0.09 (ΔT = 30 ℃)
N-Bi_2Te_3+P-Sb_2Te_3 [52]	~0.1 (ΔT = 20 ℃)
N-Bi_2Te_3(PEDOT:PSS)+P-Sb_2Te_3(PEDOT:PSS) [53]	~0.2 (ΔT = 20 ℃)
Bi_2Te_3 with 1 wt% Se single leg [54]	~0.14 (ΔT = 20 ℃)
P-Sb + N-Ni [55]	~0.9 (ΔT = 20 ℃)
N-Bi_2Te_3+P-Sb_2Te_3 [56]	~1.42 (ΔT = 60 ℃)
Ag_2Se single leg [36]	~0.16 (ΔT = 20 ℃)
Ag_2Se single leg [29]	~0.23 (ΔT = 30 ℃)
Ag_2Se single leg [21]	~1.0 (ΔT = 20 ℃)
Ag_2Se single leg [38]	~1.4 (ΔT = 20 ℃)
This work	1.5 (ΔT = 20 ℃)

Supplementary Table 4. A summary of reported surface energies of other materials.

**Materials**	**Surface Energy (J m^{-2})**
Mg_2Si (100) [57]	0.8-2.0
Al_3Ti (010) [58]	1.798
Al_3Ti (001) [58]	2.374/1.167
Boron Carbide Polytype $B_{11}C_p$(CBC) ($10\bar{1}1$) [59]	3.21
Co (0001) [60]	2.110
Co_3Cr (0001) [60]	2.170
Co_3Mn (0001) [60]	2.766
Co_3Ni (0001) [60]	2.012
$CoSb_3$ (100) [61]	0.330
$CoSb_3$ (110) [61]	0.138
$CoSb_3$ (111) [61]	0.797
Ni_3Nb (100) [62]	2.51
Ti_2AlC (0001) [63]	0.078
This work Ag_2Se (00*l*)	0.032-0.064<br>(0.002 eV/Å^2-0.004 eV/Å^2)

References (Supplementary Information)

1. Xu, Y. et al. Performance optimization and single parabolic band behavior of thermoelectric MnTe. *J. Mater. Chem. A* **5**, 19143-19150 (2017).

2. Shen, J., Chen, Z., Zheng, L., Li, W. & Pei, Y. Single parabolic band behavior of thermoelectric *p*-type $CuGaTe_2$. *J. Mater. Chem. C* **4**, 209-214 (2016).

3. She, X. et al. High thermoelectric performance of higher manganese silicides prepared by ultra-fast thermal explosion. *J. Mater. Chem. C* **3**, 12116-12122 (2015).

4. Liu, W. et al. Ag Doping Induced Abnormal Lattice Thermal Conductivity in Cu_2Se. *J. Mater. Chem. C* **6**, 13225-13231 (2018).

5. Kresse, G.Hafner, J. *Ab initio* molecular-dynamics simulation of the liquid-metal-amorphous-semiconductor transition in germanium. *Phys. Rev. B* **49**, 14251-14269 (1994).

6. Kresse, G.Hafner, J. Ab initio molecular dynamics for liquid metals. *Phys. Rev. B* **47**, 558-561 (1993).

7. Kresse, G.Furthmüller, J. Efficiency of ab-initio total energy calculations for metals and semiconductors using a plane-wave basis set. *Comp. Mater. Sci.* **6**, 15-50 (1996).

8. Kresse, G.Hafner, J. Norm-conserving and ultrasoft pseudopotentials for first-row and transition elements. *J. Phys. Condens. Mat.* **6**, 8245-8257 (1994).

9. Kresse, G.Furthmüller, J. Efficient iterative schemes for ab initio total-energy calculations using a plane-wave basis set. *Phys. Rev. B* **54**, 11169-11186 (1996).

10. Kresse, G.Joubert, D. From ultrasoft pseudopotentials to the projector augmented-wave method. *Phys. Rev. B* **59**, 1758-1775 (1999).

11. Perdew, J.P., Burke, K. & Ernzerhof, M. Generalized Gradient Approximation Made Simple. *Phys. Rev. Lett.* **77**, 3865-3868 (1996).

12. Cococcioni, M.de Gironcoli, S. Linear response approach to the calculation of the effective interaction parameters in the LDA + U method. *Phys. Rev. B* **71**, 035105 (2005).

13. Setyawan, W.Curtarolo, S. High-throughput electronic band structure calculations: Challenges and tools. *Comp. Mater. Sci.* **49**, 299-312 (2010).

14. Tran, F.Blaha, P. Accurate Band Gaps of Semiconductors and Insulators with a Semilocal Exchange-Correlation Potential. *Phys. Rev. Lett.* **102**, 226401 (2009).

15. Andrés, J., Gracia, L., Gouveia, A.F., Ferrer, M.M. & Longo, E. Effects of surface stability on the morphological transformation of metals and metal oxides as investigated by first-principles calculations. *Nanotechnology* **26**, 405703 (2015).

16. Jiang, C. et al. Ultrahigh Performance of *n*-Type Ag_2Se Films for Flexible Thermoelectric Power Generators. *ACS Appl. Mater. Interfaces* **12**, 9646-9655 (2020).

17. Gao, Q. et al. High Power Factor Ag/Ag_2Se Composite Films for Flexible Thermoelectric Generators. *ACS Appl. Mater. Interfaces* **13**, 14327-14333 (2021).

18. Zhou, K. et al. Non-epitaxial pulsed laser deposition of Ag_2Se thermoelectric thin films for near-room temperature applications. *Ceram. Int.* **42**, 12490-12495 (2016).

19. Lu, Y. et al. Ultrahigh performance PEDOT/Ag_2Se/CuAgSe composite film for wearable thermoelectric power generators. *Mater. Today Phys.* **14**, 100223 (2020).

20. Zheng, Z.-H. et al. Realizing high thermoelectric performance in highly (0*l*0)-textured flexible Cu_2Se thin film for wearable energy harvesting. *Mater. Today Phys.* **24**, 100659 (2022).

21. Lei, Y. et al. Microstructurally Tailored Thin β-Ag_2Se Films towards Commercial Flexible Thermoelectrics. *Adv. Mater.* **34**, 2104786 (2021).

22. Jiang, C. et al. Ultrahigh performance polyvinylpyrrolidone/Ag_2Se composite thermoelectric film for flexible energy harvesting. *Nano Energy* **80**, 105488 (2021).

23. Saeidi-Javash, M. et al. Machine learning-assisted ultrafast flash sintering of high-performance

and flexible silver–selenide thermoelectric devices. *Energy Environ. Sci.* **15**, 5093-5104 (2022).

24. Mallick, M.M. et al. New frontier in printed thermoelectrics: formation of β-Ag_2Se through thermally stimulated dissociative adsorption leads to high *ZT*. *J. Mater. Chem. A* **8**, 16366-16375 (2020).

25. Mallick, M.M., Franke, L., Rösch, A.G. & Lemmer, U. Shape-Versatile 3D Thermoelectric Generators by Additive Manufacturing. *ACS Energy Lett.* **6**, 85-91 (2021).

26. Li, Y. et al. Exceptionally High Power Factor Ag_2Se/Se/Polypyrrole Composite Films for Flexible Thermoelectric Generators. *Adv. Funct. Mater.* **32**, 2106902 (2022).

27. Palaporn, D., Mongkolthanaruk, W., Faungnawakij, K., Kurosaki, K. & Pinitsoontorn, S. Flexible Thermoelectric Paper and Its Thermoelectric Generator from Bacterial Cellulose/Ag_2Se Nanocomposites. *ACS Appl. Energy Mater.* **5**, 3489-3501 (2022).

28. Yang, Z.-y. et al. Ag_2Se/nylon self-supporting composite films for wearable photo-thermoelectric generators with high output characteristics. *J. Mater. Chem. A* **10**, 21080-21092 (2022).

29. Ding, Y. et al. High Performance n-Type Ag_2Se Film on Nylon Membrane for Flexible Thermoelectric Power Generator. *Nat. Commun.* **10**, 841 (2019).

30. Zheng, Z.-H. et al. Significantly (00*l*)-textured Ag_2Se thin films with excellent thermoelectric performance for flexible power applications. *J. Mater. Chem. A* **10**, 21603-21610 (2022).

31. Mallick, M.M. et al. High-Performance Ag–Se-Based n-Type Printed Thermoelectric Materials for High Power Density Folded Generators. *ACS Appl. Mater. Interfaces* **12**, 19655-19663 (2020).

32. Lee, D. et al. Substrate-Free Thermoelectric 25 μm-Thick Ag_2Se Films with High Flexibility and In-Plane *zT* of 0.5 at Room Temperature. *ACS Appl. Mater. Interfaces* **15**, 3047-3053 (2023).

33. Perez-Taborda, J.A., Caballero-Calero, O., Vera-Londono, L., Briones, F. & Martin-Gonzalez, M. High Thermoelectric *zT* in n-Type Silver Selenide films at Room Temperature. *Adv. Energy Mater.* **8**, 1702024 (2018).

34. Manzano, C.V., Llorente del Olmo, C., Caballero-Calero, O. & Martín González, M. High thermoelectric efficiency in electrodeposited silver selenide films: from Pourbaix diagram to a flexible thermoelectric module. *Sustain. Energy Fuels* **5**, 4597-4605 (2021).

35. Gao, J. et al. Thermoelectric Flexible Silver Selenide Films: Compositional and Length Optimization. *iScience* **23**, 100753 (2020).

36. Lu, Y. et al. Ultrahigh power factor and flexible silver selenide-based composite film for thermoelectric devices. *Energy Environ. Sci.* **13**, 1240-1249 (2020).

37. Sarkar, P. et al. Improved Power Factor in Highly Textured n-Type Ag_2Se Flexible Films. *ACS Appl. Electron. Mater.* **5**, 1650-1659 (2023).

38. Hou, S. et al. High-performance, thin-film thermoelectric generator with self-healing ability for body-heat harvesting. *Cell Rep. Phys. Sci.* **3**, 101146 (2022).

39. Xin, C. et al. Solution-Processed Flexible n-Type S-Doped Ag_2Se Thermoelectric Generators for Near-Ambient-Temperature Energy Harvest. *Mater. Today Energy* **33**, 101266 (2023).

40. Liu, Y. et al. High performance Ag_2Se films by a one-pot method for a flexible thermoelectric generator. *J. Mater. Chem. A* **10**, 25644-25651 (2022).

41. Hu, Q.-X. et al. SWCNTs/Ag_2Se film with superior bending resistance and enhanced thermoelectric performance *via in situ* compositing. *Chem. Eng. J.* **457**, 141024 (2023).

42. Hou, S. et al. High performance wearable thermoelectric generators using Ag_2Se films with large carrier mobility. *Nano Energy* **87**, 106223 (2021).

43. Niu, J. et al. *In-situ* growth of high room temperature thermoelectric performance Ag_2Se thin films. *Mater. Lett.* **312**, 131662 (2022).

44. Wang, Z. et al. High performance Ag_2Se/Ag/PEDOT composite films for wearable thermoelectric power generators. *Mater. Today Phys.* **21**, 100553 (2021).

45. Geng, J. et al. High Power Factor n-Type Ag_2Se/SWCNTs Hybrid Film for Flexible

Thermoelectric Generator. *J. Phys. D Appl. Phys.* **54**, 434004 (2021).

46. Lu, Y., Liu, Y., Li, Y. & Cai, K. The influence of Ga doping on preparation and thermoelectric properties of flexible Ag_2Se films. *Compos. Commun.* **27**, 100895 (2021).

47. Jindal, S., Singh, S., Saini, G.S.S. & Tripathi, S.K. Optimization of thermoelectric power factor of (013)-oriented Ag_2Se films *via* thermal annealing. *Mater. Res. Bull.* **145**, 111525 (2022).

48. Wu, M. et al. Ultraflexible and High-Thermoelectric-Performance Sulfur-Doped Ag_2Se Film on Nylon for Power Generators. *ACS Appl. Mater. Interfaces* **14**, 4307-4315 (2022).

49. Park, D., Lee, S. & Kim, J. Thermoelectric and mechanical properties of PEDOT:PSS-coated Ag_2Se nanowire composite fabricated *via* digital light processing based 3D printing. *Compos. Commun.* **30**, 101084 (2022).

50. Park, D., Ju, H. & Kim, J. Enhanced thermoelectric properties of flexible N-type Ag_2Se nanowire/polyvinylidene fluoride composite films synthesized *via* solution mixing. *J. Ind. Eng. Chem.* **93**, 333-338 (2021).

51. Lu, Y. et al. Good Performance and Flexible PEDOT:PSS/Cu_2Se Nanowire Thermoelectric Composite Films. *ACS Appl. Mater. Interfaces* **11**, 12819-12829 (2019).

52. Yu, Y. et al. Towards high integration and power density: Zigzag-type thin-film thermoelectric generator assisted by rapid pulse laser patterning technique. *Appl. Energ.* **275**, 115404 (2020).

53. We, J.H., Kim, S.J. & Cho, B.J. Hybrid Composite of Screen-Printed Inorganic Thermoelectric Film and Organic Conducting Polymer for Flexible Thermoelectric Power Generator. *Energy* **73**, 506-512 (2014).

54. Madan, D. et al. Enhanced Performance of Dispenser Printed MA n-type Bi_2Te_3 Composite Thermoelectric Generators. *ACS Appl. Mater. Interfaces* **4**, 6117-6124 (2012).

55. Cao, J. et al. Flexible elemental thermoelectrics with ultra-high power density. *Mater. Today Energy* **25**, 100964 (2022).

56. Ao, D.-W. et al. Assembly-Free Fabrication of High-Performance Flexible Inorganic Thin-Film Thermoelectric Device Prepared by a Thermal Diffusion. *Adv. Energy Mater.* **12**, 2202731 (2022).

57. Liao, J.N., Li, K., Wang, F., Zeng, X.S. & Zhou, N.G. Properties of Mg_2Si (100) surfaces: A first-principles study. *Solid State Commun.* **183**, 41-46 (2014).

58. Qin, J. Partition the total energy of Al_3Ti to characterize the Al_3Ti/Al interface properties: a first-principles study. *Surf. Sci.* **739**, 122398 (2024).

59. Beaudet, T.D., Smith, J.R. & Adams, J.W. Surface energy and relaxation in boron carbide $(10\bar{1}1)$ from first principles. *Solid State Commun.* **219**, 43-47 (2015).

60. Cao, Y., Lin, Q., Huang, Q., Xu, Y. & Zhou, S. First-principles predictions of corrosion resistance of (0001) surface of Co and Co_3X (X = Cr, Ni, Mn) compounds. *Computational and Theoretical Chemistry* **1225**, 114171 (2023).

61. Hammerschmidt, L., Quennet, M., Töpfer, K. & Paulus, B. Low-index surfaces of $CoSb_3$ skutterudites from first principles. *Surf. Sci.* **637-638**, 124-131 (2015).

62. Hao, L., Chen, W., Lei, X., Yao, W. & Wang, N. Structures, Energies, and Electronic Properties of Low-Index Surfaces of γ''-Ni_3Nb: A First-Principles Calculations. *physica status solidi (b)* **260**, 2300239 (2023).

63. Liu, P. et al. A systematic investigation on the surface properties of Ti_2AlC *via* first-principles calculations. *Surf. Sci.* **735**, 122337 (2023).
